# Time-dependent microbial shifts during crayfish decomposition in freshwater and sediment under different environmental conditions

**DOI:** 10.1038/s41598-023-28713-x

**Published:** 2023-01-27

**Authors:** Bastian Mähler, Kathrin Janssen, Mara Iris Lönartz, Markus Lagos, Thorsten Geisler, Jes Rust, Gabriele Bierbaum

**Affiliations:** 1 Section Paleontology, Institute of Geosciences, Rheinische Friedrich-Wilhelms Universität Bonn, 53115 Bonn, Germany; 2 Institute of Medical Microbiology, Immunology and Parasitology, Medical Faculty, Rheinische Friedrich-Wilhelms Universität Bonn, 53127 Bonn, Germany; 3 Section Geochemistry, Institute of Geosciences, Rheinische Friedrich-Wilhelms-Universität Bonn, 53115 Bonn, Germany; 4 Institute of Energy and Climate Research (IEK-6): Nuclear Waste Management, Forschungszentrum Jülich GmbH, 52428 Jülich, Germany

**Keywords:** Palaeontology, Microbiology

## Abstract

Fossilization processes and especially the role of bacterial activity during the preservation of organic material has not yet been well understood. Here, we report the results of controlled taphonomic experiments with crayfish in freshwater and sediment. 16S rRNA amplicon analyzes showed that the development of the bacterial community composition over time was correlated with different stages of decay and preservation. Three dominating genera, *Aeromonas*, *Clostridium* and *Acetobacteroides* were identified as the main drivers in the decomposition of crayfish in freshwater. Using micro-computed tomography (µ-CT), scanning electron microscopy (SEM) and confocal Raman spectroscopy (CRS), calcite clusters were detected after 3–4 days inside crayfish carcasses during their decomposition in freshwater at 24 °C. The precipitation of calcite clusters during the decomposition process was increased in the presence of the bacterial genus *Proteocatella*. Consequently, *Proteocatella* might be one of the bacterial genera responsible for fossilization.

## Introduction

“Konservat-Lagerstätten” harbor a distinct soft tissue fossil record and, thereby, depict the diversity of ancient communities^1,2^. The different steps that lead to fossilization of soft tissues remain unclear and, especially, the role of bacterial activity is not well understood. Immediately after the death of an organism, autolytic enzymes, such as lipases, proteases or amylases, start self-digestion of the dead cells and nutrient-rich liquids are released^3–6^. These nutrients support the initial growth of bacteria that then produce hydrolytic exoenzymes and continue to decompose the organic matter. Heterotrophic decay may be accompanied by shifts in pH-value. It is assumed that these pH shifts could then result in the release of ions from the organic material and the surrounding environment. Free ions further impair bacterial growth, lead to a stabilization of tissue remains and—eventually—to authigenic mineralization^7–13^. Therefore, the initial bacterial activity seems to play an important role for exceptional preservation^14,15^ as long as the rate of subsequent preservation processes by, e.g., mineralization, remains higher than decomposition rates^7–12,14,16–20^.

Most of the research focuses on environmental conditions that either reduce bacterial activity or support the precipitation of minerals. Bacterial activity is highly influenced by abiotic factors, such as temperature, pH-value, salinity or oxygen. These parameters impact the ability of strains to successfully colonize a habitat or regulate the production of specific degrading exoenzymes according to the availability of nutrients, oxygen, bacterial cell density and bacterial growth phase^21–23^. Exoenzymes such as proteases, chitinases or lipases contribute to decomposition of soft tissue, whereas other bacterial metabolic characteristics (e.g., urease activity, amino acid metabolism, fermentation of sugars with release of organic acids) can directly or indirectly induce mineralization processes^24^, and thereby contribute to the preservation of soft tissues. It is assumed that decay and mineralization occur simultaneously and are specific to the tissue type^13,25^. Preservation of soft tissue has often been associated with anaerobic conditions that reduce autolytic activity and are assumed to influence microbial degradation^26–35^. However, this does not consider that bacteria exhibit various adaptations to reduced oxygen conditions. For instance, *Pseudomonas tunicata*, an aerobic marine bacterium will form pseudomorphs of marine embryos under aerobic conditions, but will destroy the internal structure of the cells if oxygen is not available or depleted^31^. In addition, some obligate anaerobic organisms, like sulfate-reducing bacteria, are able to degrade organic matter as fast as aerobic bacteria^16,36^. Furthermore, taphonomic experiments still did not reach a consensus on the role of anaerobic conditions on preservation. While some results indicate faster decomposition of soft tissue under aerobic conditions^26–28,37^, other experimental results show no differences in comparison to anaerobic decay^16,38^ or only rare differences under various oxygen conditions^39,40^. Especially, one study highlighted that some cnidarian tissues decayed even faster under anaerobic conditions^41^. It has also been suggested that specific mineralization processes induced by bacteria under anaerobic conditions might be more important for tissue preservation than the reduction of microbial activity^14^.

Currently, there is only very limited knowledge about the impact of individual species on decay and preservation processes and their origin, either from the animal microbiome (gastro intestinal/integumental flora) or from the biotope in which the animal died (environment). First results indicated a stabilizing influence of endogenous microorganisms presumably from the gastro-intestinal flora of bilaterian organisms^34^, whereas Eagan^35^ highlighted that each organism exhibits an individual microbiome that can either support preservation or fasten decay. Therefore, controlled and carefully designed taphonomic experiments focusing on microbial activity and the associated organic changes under different environmental conditions are necessary to obtain a detailed understanding of the decay processes. The investigation of decomposition processes offers the opportunity to identify the phases of decay that either have to be suppressed or that have to take place in order to generate the specific conditions necessary for soft tissue preservation.

**Figure 1:**
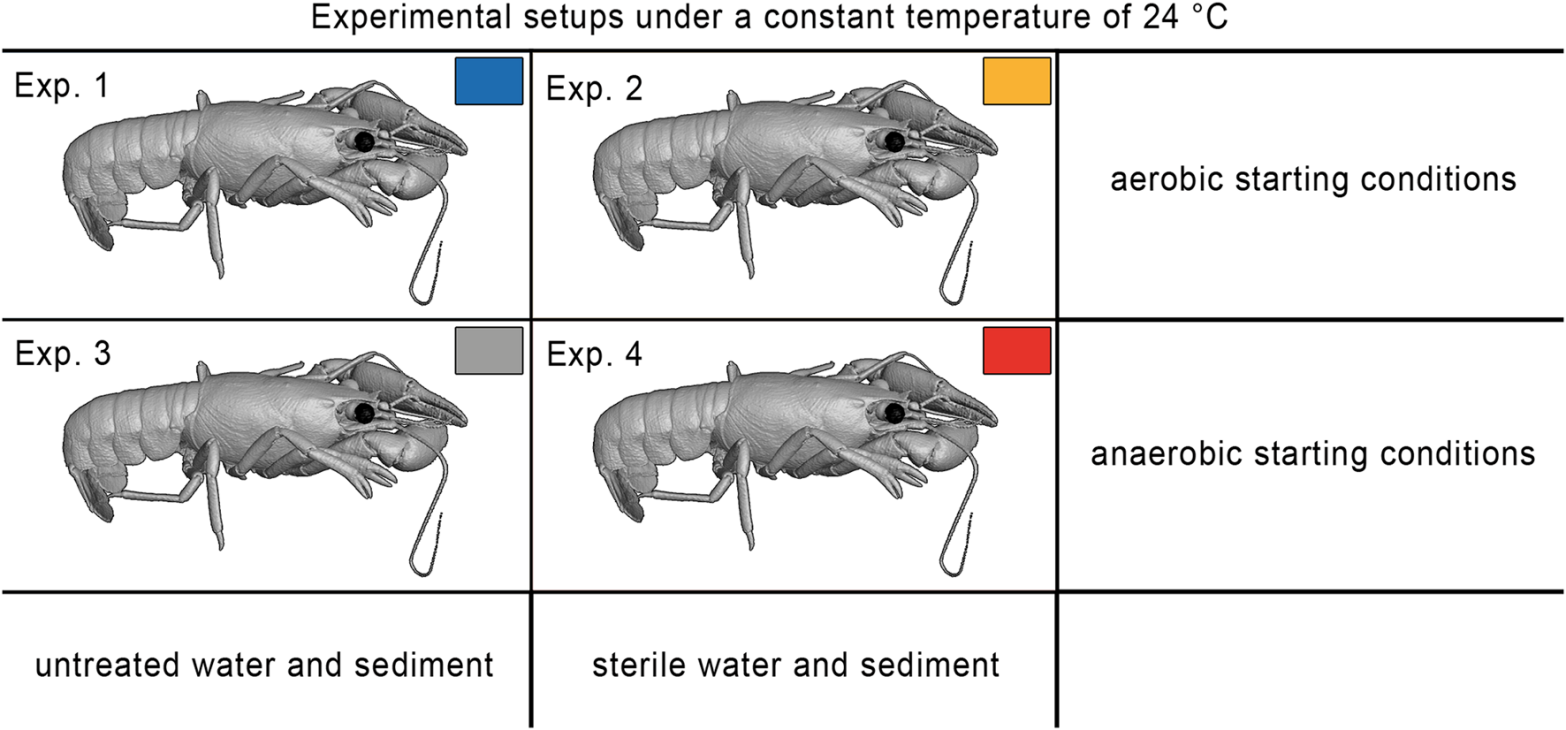
Experimental setup design conducted at a constant temperature of 24 °C. Exp. 1 was conducted under aerobic conditions with untreated water and sediment. Exp. 2 was conducted under aerobic conditions with sterile water and sediment. Exp. 3 was conducted under anaerobic conditions with untreated water and sediment. Exp. 4 was conducted under anaerobic conditions with sterile water and sediment.

With this study, we want to contribute to the elucidation of the complex interplay between decay and preservation that paves the way to an excellently preserved fossil. This is the first experimental approach that analyzed the time-dependent shift of the bacterial community composition during decay or preservation of *Cambarellus diminutus* carcasses in controlled taphonomic freshwater experiments by 16S rRNA amplicon analysis. To elucidate the role of the intrinsic and extrinsic flora on the degradation or preservation of soft tissues, the crayfish were incubated in natural lake water and sediment at 24 °C. Autoclaved lake water and sediment served as controls. Anaerobic and aerobic conditions were used to study the influence of anoxia on the decay and potential preservation processes (Fig. 1).

## Methods

### Study design and sample preparation

#### Animal selection

Individuals of the extant crayfish *Cambarellus diminutus* [Hobbs 1945] were taken from a breeding tank community raised in our lab. The animals were kept in 54 L tanks of 60 × 30 × 30 cm in size, at a constant water temperature of 26 °C. Tanks were filled with pipe water and fortified with “Biotopol C” water conditioner (JBL, GmbH & Co. KG, Neuhofen, Germany) to neutralize zinc (Zn) and lead (Pb) and to remove chlorine (Cl) and bind copper (Cu). The crayfish were fed with nothing but “Crabs Nature” (Sera, GmbH, Heinsberg, Germany), a main food for crayfish (ingredients can be found in Supplementary Table 1.

144 individuals with partly filled guts were sacrificed by placing them in an atmosphere of carbon dioxide (CO_2_). Specimens were not dried before weighing on a micro scale. Lengths were measured from the anterior tip of the cephalothorax to the end of the pleon without the telson (Supplementary Table 2a,b). Information about the systematic zoological position and the general construction of the cuticle of *Cambarellus diminutus* and the moulting cycle are provided in the supplementary information (Supplementary Figs. 1 and 2).

#### Experimental setup

In the study four different experimental setups were conducted at a constant temperature of 24 °C (Fig. 1 and Supplementary Fig. 3). Two experiments were started with aerobic conditions, whereby one experiment was conducted with untreated lake water and sediment (Exp. 1) and the other with sterile water and sediment (Exp. 2). Two further experiments were also performed with untreated (Exp. 3) and sterile sediment and water (Exp. 4), but anaerobic conditions were established through specific gas generating systems (Fig. 1).

**Figure 2:**
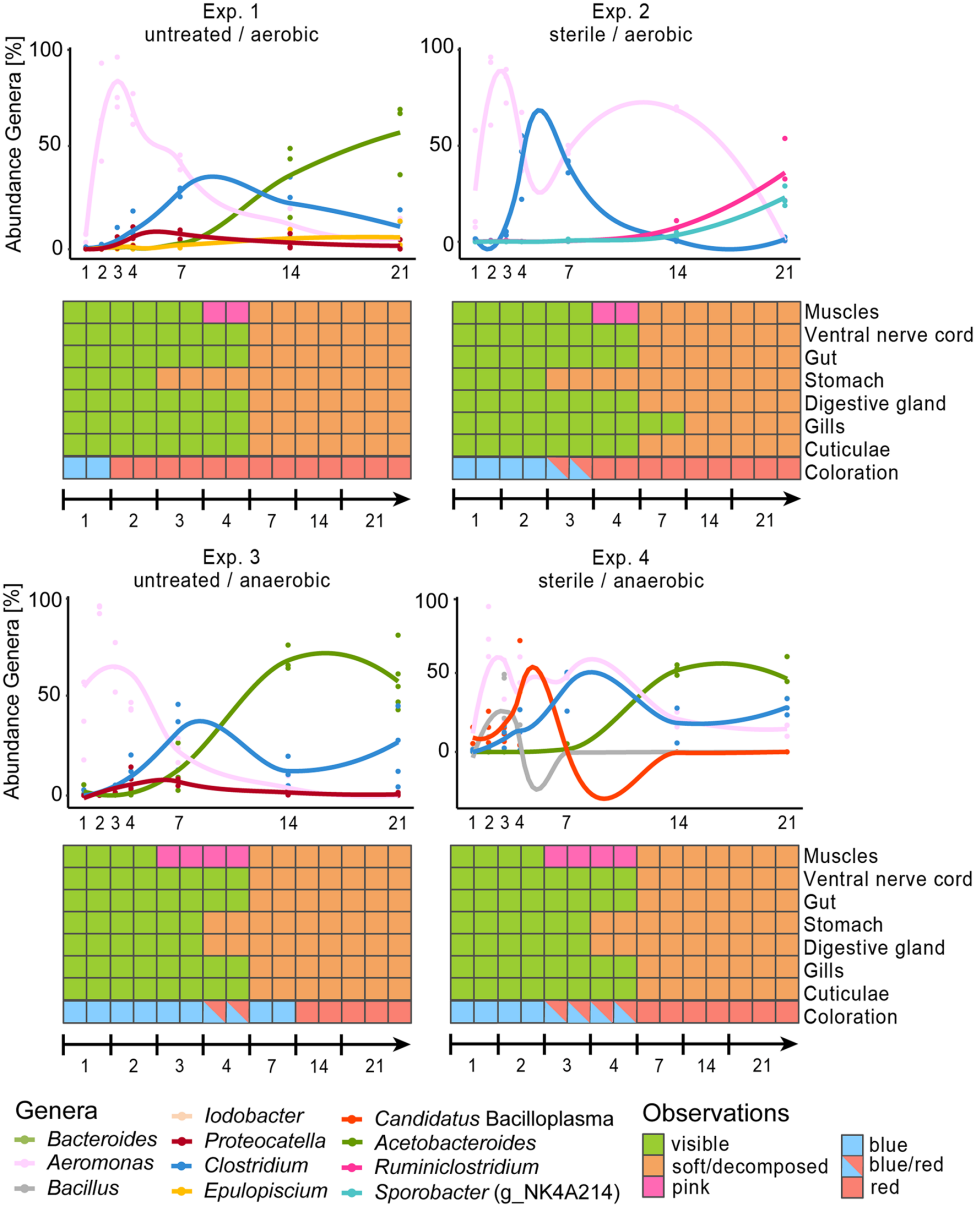
Time-dependent abundance of dominant genera and optical decay of body areas and inner organs of decaying crayfish for a duration of 21 days at 24 °C.

**Figure 3:**
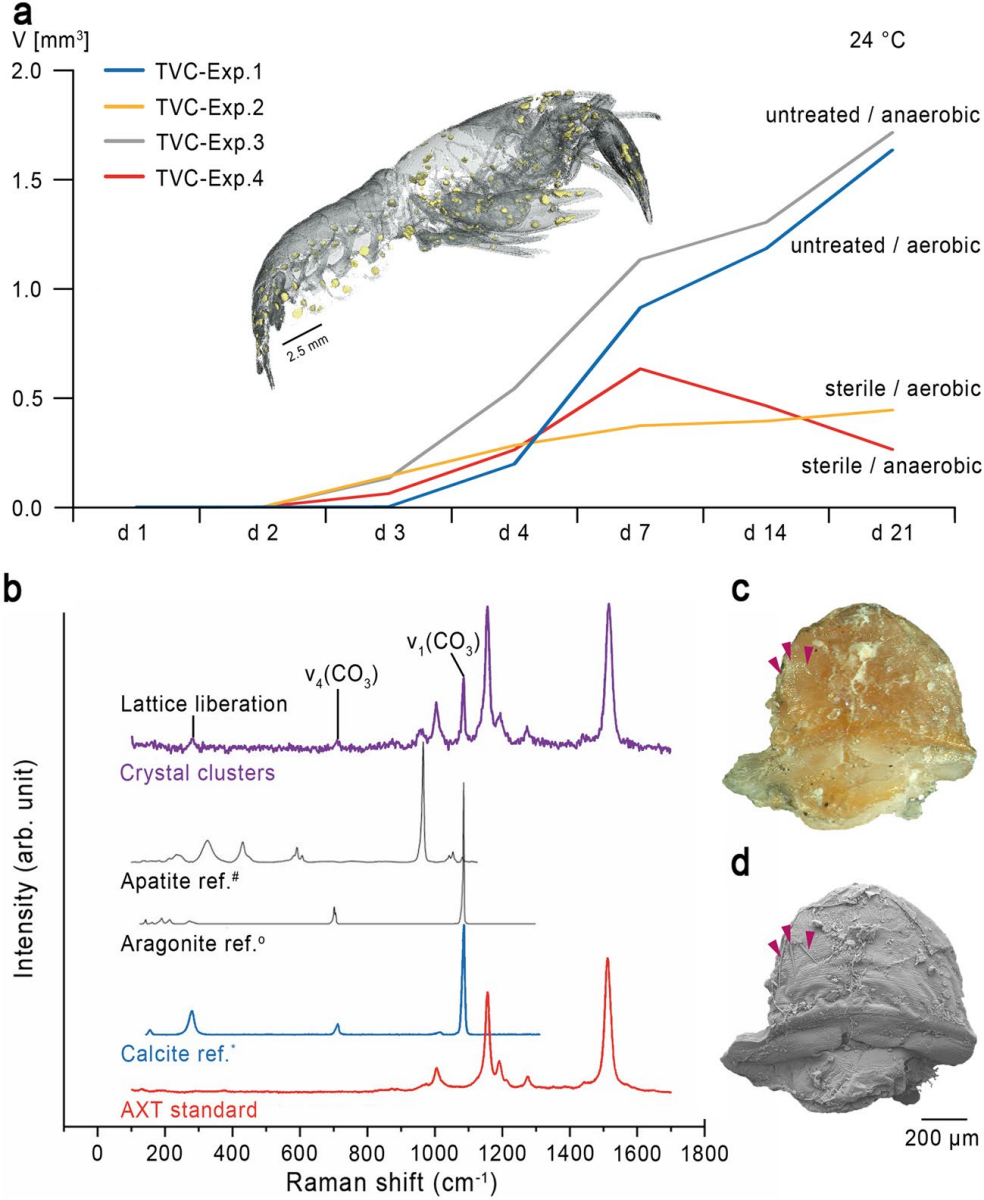
Calcite precipitation inside decomposing crayfish. (**a**) Median value of the increase in the total volume of calcite of Exp. 1–4 for a duration of 21 days at a constant temperature of 24 °C, including a translucent 3D-model of an individual showing reconstructed 3D-models of calcite clusters at the inner side of the cuticle (Yellow structures). (**b**) Representative Raman spectra of observed crystal clusters compared to Raman reference spectra of crystalline apatite, aragonite and calcite, taken from the RRUFF Raman data base (#R060070, 0R060070, *R040170^42^). Raman spectra of the crystal clusters exhibit main Raman bands typically for crystallized calcite and the β-carotene, astaxanthin. (**c**) Optical image of a semi-circular calcite cluster with three mineralized setae (pink arrows). (**d**) SEM image of the calcite cluster shown in (**c**).

For all experiments 184 sterile Falcon tubes (50 mL) were filled with ~ 6 g of near shore sediment, taken from a freshwater lake called “Alte Tongrube” in Bonn-Röttgen, Germany (50°40′24.7″N/7°04′29.6″E). 144 specimens were each placed on the sediment in one of these Falcon tubes and 40 Falcon tubes remained without a crayfish specimen and served as blank samples. All tubes were filled with ~ 40 mL of lake water, taken from the same locality. For sterile experiments (Exp. 2 and 4) the water and sediment were autoclaved for at least 20 min at 121 °C. All experiments were set up under a laminar flow hood. Experiments under anaerobic starting conditions (Exp. 3 and 4) were prepared with the addition of 10 mM β-mercaptoethanol (Sigma-Aldrich Chemie GmbH, GER) as a reducing agent. Anaerobic conditions were established with gas generating systems BD Difco™ GasPak™ EZ Anaerobier Pouch System (BD Becton, Dickinson and Company, USA). Each experiment had seven sampling days (day 1, 2, 3, 4, 7, 14, 21) at which morphological changes of two animals were analyzed. Attention was paid to the color of the cuticle, gas accumulation and disarticulations. The specimens were then dissected and examined with a stereomicroscope (Stemi 2000; Carl Zeiss Microscopy Deutschland GmbH, Oberkochen, Germany). The focus of analysis during the dissection was set on internal changes of the following organs: gills, digestive gland, stomach, gut, ventral nerve cord and muscle tissue (Fig. 2). The image of the calcite conglomerate in Fig. 3 was photographed with a stereo-zoom-microscope (Axio Zoom. V16, Carl Zeiss Microscopy Deutschland, Oberkochen, Germany). Final figures were created by using Adobe Photoshop CS5 (Adobe, Dublin, Republic of Ireland) with 300 dpi.

Three other crayfish specimens were used for DNA extraction to analyze the microbiological changes. To monitor the changes in the microbiome and the surrounding water and sediment in the absence of crayfish, blank samples without tissue were implemented on day 1, 7, 14, and 21 and all tests were performed in triplicates as controls. At the beginning of each experiment carcasses were fully articulated and blue in color. In addition, no symbiotic, parasitic or commensally organisms were found on the carcasses.

#### DNA extraction

Prior to DNA extraction, water samples were filtered with PORAFIL^®^ NC filters (d = 0.25 µm; Macherey–Nagel GmbH & Co. KG, GER). Filters were then cut into small pieces and were transferred into ZR BashingBead™ Lysis Tubes (0.1 and 0.5 mm) with 750 µL ZymoBIOMICS Lysis solution. Bead beating of filters and tissue samples was performed with Precellys^®^ homogenizer (Bertin Technologies S.A.S., Montigny Le Bretonneux, FR), 6000×*g* for 30 s. DNA was extracted with the ZymoBIOMICS DNA/RNA Miniprep Kit (Zymo Research, USA) according to the manufacturer’s instructions. DNA was eluted in 50 µL DNase/RNase-free water and the DNA concentration in ng per µL was determined by a NanoDrop™ OneC Microvolume-UV/VIS spectrophotometer (Thermo Fisher Scientific Inc., USA).

#### 16S rRNA gene amplicon analysis

For 16S rRNA gene sequencing, the V4 variable region of the 16S rRNA gene sequence was amplified with the specific 16S primers of 16s-515F (GTG CCA GCM GCC GCG GTA A) and 16s-806R (GGA CTA CVS GGG TAT CTA AT)^43^. The PCR reaction was performed as a single-step PCR with the HotStarTaq Plus Master Mix Kit (Qiagen, USA) and included an initial denaturation at 95 °C for 5 min, followed by 30–35 cycles of 95 °C for 30 s, 53 °C for 40 s, and 72 °C for 1 min, with a final elongation step at 72 °C for 10 min. Paired end sequencing (bTEFAP^®^) was performed by Molecular Research DNA (http://www.mrdnalab.com, Shallowater, USA) on a MiSeq following the manufacturer’s guidelines^44^. Raw sequence data was processed via the QIIME2^45^ with default parameters unless otherwise noted. DADA2 pipeline was used for sequence quality control, denoising, and chimeric filtering^46^. Taxonomy classification of the final ASVs (amplicon sequencing variant), clustered at 99% identity, was performed with a naive Bayesian classifier that was trained against SILVA database release 138 especially for 515F/806R rRNA region^47,48^.

#### Isolation of bacterial strains

Each sampling day, water and tissue samples were prepared for cultivation of bacterial species. In brief, the surface of the crayfish tissue was swabbed for isolation of biofilm forming species. The swab was incubated in tryptone soy broth (TSB) medium plus 1% glucose in ten different dilutions for 3 days at 30 °C and then plated on Columbia agar with 5% sheep blood (COL [BD™, Becton, Dickinson and Company, New Jersey, USA]) for further cultivation. Additionally, 50 µL of the water samples were streaked on selective agar plates (e.g., R-2A [Sigma-Aldrich, St. Louis, USA], MacConkey agar [BD™, Becton, Dickinson and Company, New Jersey, USA], minimal LB-medium [2.5 g/L tryptone, 2.5 g/L NaCl, 1.25 g/L yeast extract, 18 g/L agar, pH 7.5]) and incubated up to 1 week at 30 °C. Further isolation was performed on COL agar. Pure cultures were identified via matrix-assisted laser desorption/ionization-time of flight mass spectroscopy (Microflex^®^ LT MALDI-TOF/MS Bruker Corporation, Billerica, USA) or 16S rRNA gene sequencing. The sequencing was performed by GATC Biotech AG (GER) and sequences were compared to EZBioCloud database^49^.

#### Bioinformatics and statistic

Analysis of alpha diversity metrics were performed using the R package “vegan” (version 2.5.6^50^. Visualizations of the statistics and microbial community composition were performed with R package “ggplot2”^51^.

#### Micro-computed tomography (µ-CT)

During the first 4 days, three specimens of Exp. 1 to 4 (E1-21.1 to E1-21.3; E2-21.1 to E2-21.3; E3-21.1 to E3-21.3; E4-21.1 to E4-21.3) were initially scanned once per day, followed by scans after 7, 14 and 21 days using a phoenix|x-ray v|tomex s 240 micro-computed-tomography (µ-CT) scanner (GE Measurement & Control, GER) located at the Institute of Geosciences of the University of Bonn. Each data set has a resolution of 38 µm; the scans were carried out at 80 kV and 100 µA. Three frames per projection were acquired by a timing of 500 ms for a total of 1000 projections. µ-CT data were processed using the software VG Studio Max 3.2 (Volume Graphics, Heidelberg, Germany [https://www.volumegraphics.com/de/produkte/vgsm/whats-new-in-vgstudio-max-3-2-x.html]) and Avizo 8.0 (Thermo Fisher Scientific, Schwerte, Germany [https://www.thermofisher.com/de/de/home/electron-microscopy/products/software-em-3d-vis/avizo-software.html]) to reconstruct and visualize precipitated crystal clusters inside the specimens, and gastroliths located inside the stomach. In addition, Avizo 8.0 was used for volume measurements of polygonal 3D-surface models.

#### Confocal Raman spectroscopy (CRS)

Crystal clusters (if present) were obtained from the carcasses and analyzed with a LabRam HR800 confocal Raman spectrometer (Horiba Scientific) located at the Institute of Geosciences of the University of Bonn. A 100 mW 784 nm diode laser as excitation source, a grating of 600 grooves/mm, and a 100 × objective with a numerical aperture of 0.9 were used. The confocal hole size and the spectrometer entrance slit size were set to 1000 and 100 µm, respectively. With these settings the spectral resolution was 4.6 cm^−1^. The total exposure time was 2 min with four accumulations of 30 s.

#### Scanning electron microscopy (SEM)

Crystal clusters (if present) were dissected and coated by a thin layer of gold with a cool sputter coater (Cressington Sputter Coater 108 manual, Tescan GmbH, Dortmund, Germany). Samples were subsequently scanned with an ‘environmental’ scanning electron microscope (SEM) unit (TESCAN VEGA 4 LMU) by using the SE detector at 20 keV. Images have 1536 × 1331 pixel and 16 bit. The working distance of each SEM image can be found in the figure captions.

## Results

### General observations on the decay process

In untreated lake water under aerobic conditions (Exp. 1) a color change of the cuticles and a strong odor of decay were observed on day 2. Comparable observations were made in sterilized lake water under aerobic (Exp. 2) and anaerobic conditions (Exp. 4) after 3 days and also after 4 days under anaerobic conditions in untreated lake water (Exp. 3) (Fig. 2 and Supplementary Table 3). On day 7 crayfish cuticles appeared soft and jellylike in all experiments.

Under anaerobic conditions (Exp. 3 and 4), regardless of the treatment of the water, the initially white muscles showed a pink discoloration on day 3, whereas the same changes were noticed on day 4 in experiments that started under aerobic conditions (Exp. 1 and 2).

In all individuals of all experiments, the stomach decomposed within the first 4 days (Fig. 2), as well as the digestive gland in the individuals, which had been incubated under anaerobic conditions. Under aerobic conditions, the digestive gland could be identified for at last 1 week. After this time, muscles were pulpy and cuticles were translucent and jellylike. The ventral nerve cord, gut and gills were no longer visible in any of the animals, with the exception of the gills in individuals of Exp. 2 (aerobic, sterile) (Fig. 2), which were visible up to day 14.

After 2 weeks, the sediment and the carcasses of Exp. 1 to 3 were covered by a black layer that appeared in Exp. 4 (anaerobic, sterile) only after 21 days. In addition, an accumulation of putrefaction gas was noticed around the gill area in individuals of Exp. 1 (aerobic, untreated), which resulted in a detached floating cephalothorax of individual E1-21.1 on day 24, 3 days after the regular study period (Supplementary Fig. 4a). The cephalothorax of individual E1-21.3 was only partly detached from the pleon and floated, holding the remains of the dangling body, also on day 24 (Supplementary Fig. 4b).

No gas accumulation was noticed in any specimen of the other experiments, but we observed a separation of the pleon from the cephalothorax in individuals of Exp. 2 (aerobic, sterile) after 7 days and in crayfish specimens of Exp. 3 (anaerobic, untreated) after 2 weeks.

### Time-dependent shifts of the microbial community

To analyze the temporal succession of microbial community composition (MCC) in the four experiments, we performed 16S rRNA amplicon sequencing at seven timepoints with three independent biological replicates. The bacterial succession within the triplicates was comparable and allowed an interpretation of distinct tendencies. The MCC of the sediment was stable throughout the seasons (Supplementary Table 4). The α-diversity, calculated via the Shannon index^52^, decreased under all conditions within the first 3 days. In the control experiments (Exp. 2: aerobic, sterile; Exp. 4: anaerobic, sterile), the diversity remained stable around a Shannon index of ~ 1.5, whereas in the experiments with the natural lake water a slight increase could be detected until the end of the experiments (c.f., Supplementary Table 5; Supplementary Fig. 5). In all experiments, the samples of the initial days were characterized by high amounts of Proteobacteria, but after 4 days, the phyla Firmicutes and Bacteroidetes became dominant (c.f., Supplementary Table 6). After residual oxygen had been consumed, obligate anaerobic bacteria took over in all samples. The MCC in the experiments performed with untreated lake water and sediment (Exp. 1 and Exp. 3) was first dominated by *Aeromonas* sp. These bacteria were already present on the animals on day 1 (Supplementary Table 7). The aeromonads were outcompeted by *Clostridium* sp. during the middle part of the experiment. After the stomach and digestive gland of the crayfish individuals had collapsed, species of the genus *Acetobacteroides* increased. Low abundances of *Proteocatella* sp. were found in Exp. 1 (aerobic, untreated) and Exp. 3 (anaerobic, untreated), whereas this genus was absent in the sterile controls (Exp. 2 and Exp. 4). Here, the MCCs differed from each other. Exp. 4 (anaerobic, sterile) showed a comparable bacterial progression to Exp. 1 and Exp. 3 (both untreated), although *Aeromonas* sp. showed a biphasic bloom in both control experiments (Exp. 2 and Exp. 4), i. e., a temporary decrease that was accompanied by an increase of *Clostridium* sp. for a few days. The initial decay in Exp. 4 (anaerobic, sterile) showed the presence of *Candidatus* Bacilloplasma sp. and *Bacillus* sp., whereas the decomposing animals of Exp. 2 (aerobic, sterile) were colonized by spore-forming bacteria of the genus *Sporobacter* (Ruminococcaceae NK4A214 group) and *Ruminiclostridium* in the final samples (Fig. 2 and Supplementary Table 8 for MMC of all genera). Isolated bacterial strains and their properties are shown in Supplementary Table 9.

### Formation, localization and identification of crystal clusters

µ-CT images revealed the precipitation of crystal clusters in all experimental setups, starting in the chelipeds of the individual specimen E2-21.2 in Exp. 2 already after 2 days or after 3 days in the specimens E2-21.3, E3-21.2 and E4-21.3. After 4 days, crystal clusters had precipitated in all individuals (Supplementary Table 10a). As the decay proceeded, crystalline structures were observed at the ventral side of the cephalothorax, inside the pereiopods, inside the coxa of the pleopods, along the ventro-lateral side of the tergites, in the telson, and the uropods. In addition, µ-CT images revealed that these crystal clusters only precipitated at the inner side of the cuticles. All scanned specimens of Exp. 1 (aerobic, untreated) and Exp. 2 (aerobic, sterile) contained one pair of gastroliths. Volume measurements of polygonal 3D-surface models of crystal clusters and gastroliths showed an increase of the volume of crystal clusters and simultaneously a volume reduction of gastroliths with progressive decay (Supplementary Table 10b). Importantly, in experiments, that were conducted under sterile conditions (Exp. 2 and Exp. 4), a smaller total volume of crystal clusters was observed in comparison to experiments conducted with untreated sediment and water (Exp. 1 and Exp. 3). In addition, the total volume of crystal clusters in Exp. 4 (anaerobic, sterile) decreased before the end of the experiment (Fig. 3a).

Raman analyses clearly revealed that the crystal clusters consisted of well-ordered calcite crystals (Fig. 3b), which can be identified by the fully symmetric v_1_(CO_3_) stretching vibrational band near 1085 cm^−1^ as well as the presence of bands near 154 and 281 cm^−1^ that are assigned to calcite lattice vibrations, with the latter being absent in amorphous calcium carbonate (ACC). In addition, some Raman analyses of the crystal clusters showed a mixed spectrum with fundamental bands from crystalline calcite and the β-carotene, astaxanthin (AXT) (Fig. 3b) which was identified by the typical high intensity modes near 1157 and 1517 cm^−1^ which are assigned to the C=C and C–C stretching vibrations of the polyene chain bonds, respectively^53–55^. In comparison to the spectrum obtained from the AXT standard, a small but clear deviation in the frequency of the ~ 1517 cm^−1^ band was observed, which can presumably be linked to the structural differences in the AXT.

SEM-images showed several structures of precipitated calcite clusters (Supplementary Fig. 6a–f) varying in sizes from 100 to 900 µm at the end of the experiment. One calcite structure was found inside a pereiopod of individual E1-21.3 with mineralized cuticle remains and three plumose setae (Fig. 3c,d). Most of the calcite clusters were spherical (Supplementary Fig. 6d) or bispherical. Some crystal clusters were more or less elliptical with one or two rounded thickenings at one end (Supplementary Fig. 6c,e,f). All calcite structures showed reddish discolorations and were discovered on the inner side of the cuticles (Fig. 3c and Supplementary Fig. 6a).

## Discussion

This is the first detailed investigation of the bacterial succession in soft tissue decay and preservation of arthropods in freshwater. The microbial analyses during the decay of the crayfish *C. diminutus* under different predefined parameters showed reproducible community successions in all experiments. The facultative anaerobic bacteria that dominated the tissues at the beginning of our experiments and are able to grow in the presence as well as in the absence of oxygen, were rapidly outcompeted by obligate anaerobic organisms. Similar shifts have also been described in forensic science for terrestrial decay of mice, swine and humans^56–59^. After 7 days the bacterial flora often changes due to a decrease in nutrients and need of alternative metabolic pathways to generate energy^60^, which was detected also in Exp. 1 (aerobic, untreated), Exp. 3 (anaerobic, untreated) and Exp. 4 (anaerobic, sterile). Initially the decaying carcasses were inhabited by the phylum Proteobacteria, whereas later mainly Firmicutes and Bacteroidetes were detected (see Supplementary Table 6)^58,61,62^. The microbiome of the decaying crayfish comprised three dominating genera, *Aeromonas*, *Clostridium* and *Acetobacteroides*. In all experiments the genus *Aeromonas* colonized the carcasses during the initial phase and reached maximum counts on day 2 and 3. These facultative anaerobic water bacteria occur ubiquitously^63^ and play an important role in the decomposition of carcasses in fresh- and brackish water^64,65^. They are also pathogens, causing infections of fish and other animals, as well as of immunocompromised humans^66^. The pathogenic species *A. hydrophila*, *A. salmonicida* and *A. veronii* were isolated from the experiments and confirm the prominent role *Aeromonas* in decay of water organisms as demonstrated for fish by Lobb^62^. Many of the isolates were biofilm formers. Domination of *Aeromonas* sp. in the initial decay process can be explained by the secretion of various exoenzymes, such as specific hemolysins, proteases, chitinases or lipases which contribute to host cell lysis^62,67–71^.

After 2 or 3 days, the amount of *Clostridium* increased in all experiments concomitantly with a decrease of *Aeromonas* and reached their maximum on day 7. Clostridia are associated with anaerobic infections and decay of human and animal tissues^30,58,61,62,72^ and are commonly found in the gastrointestinal tract of various organisms and in soil^73,74^. Since they were also present in the sterile controls and could rarely be detected in the sediment (see Supplementary Table 4), they must derive from the gastrointestinal tract of the crayfish individuals or spores that were present on the body surface. This genus is also frequently detected in forensic experiments^56,58,60,61^. Some species of this genus are able to cleave collagen and hyaluronic acid and produce toxins that destroy cell membranes, resulting in the release of fresh nutrients from the dead bodies^75–78^. Therefore, a lack of nutrients may have contributed to the decrease of the aeromonads that had been colonizing the carcass in the first place. Anoxia either was part of the experimental setup in Exp. 3 and Exp. 4 or developed through microbial activity in Exp. 1 and Exp. 2^14^. Later on, in all experiments *Aeromonas* sp. and *Clostridium* sp. were outcompeted by Rikenellaceae (*Acetobacteroides* sp.) after day 7, except in Exp. 2 (aerobic, sterile) in which this genus was absent and a secondary increase of aeromonads was observed. *Acetobacteroides* spp. are strictly anaerobic and ferment polymeric sugars (glycogen), hexoses, pentoses and tryptone into acetate, CO_2_, and H_2_^79^. The increase of this genus occurred simultaneously with the collapse of the stomach and digestive gland, which is a storage organ for glycogen in crustaceans^80^ indicating that liberation of substrates by breakdown of these nutrient-rich organs might have supported proliferation of *Acetobacteroides* sp. or that this genus is a natural inhabitant of these body parts. The intestine of the oriental river prawn *Macrobrachium nipponense*, which is a species in the order Decapoda such as *C. diminutus*, harbors members of *Acetobacteroides*^81^. It is noted that also the tissue controls on day 1 exhibited related species (see Supplementary Table 7) and, therefore, the absence of *Acetobacteroides* in Exp. 2 is surprising.

Mineral precipitation is often an important step in the preservation of soft bodied organisms^82^. In all experiments, the precipitation of calcite crystals was detected at the inner side of the crayfish carapaces, though to different extents. In Exp. 1 and 3 (untreated) a higher volume of calcite precipitated, independently of the oxygen content, than in Exp. 2 and 4 (sterile) (Fig. 3a). The total volume of calcite (TVC) in Exp. 4 even decreased on day 7, which can be explained by an acidification of the pH through microbial activity inside the specimens^83^. The pH was found to depend on the substrate that is degraded and may change according to the active metabolic pathways in the sample and, in turn, to influence the local chemical environment and mineral precipitation in taphonomic experiments^39,84,85^. For crayfish it was observed that the precipitation of calcite depends on the body size and/or the stage of the current moulting phase^83^. At the beginning of each moulting phase, calcium ions from the cuticle are dissolved out of the cuticle layers and are transported via the haemolymphatic circulatory system inside the cardiac stomach wall and calcium storages nodules called “gastroliths” are formed, which consist of a network of protein-chitin fibers and amorphous calcium carbonate (ACC)^86,87^. These calcium ions are therefore only available to a limited extent for calcite precipitation after death^83^. However, since the compared individuals rarely differed in body size (see Supplementary Table 2a,b) and moulting stages (Supplementary Table 10b), these factors cannot explain the significant differences in calcite precipitation.

The results of the microbiome sequencing revealed the presence of the genus *Proteocatella* in the samples of Exp. 1 and Exp. 3 in untreated lake water, whereas it was absent in Exp. 2 and Exp. 4 (sterile controls). So far, only the type species, *Proteocatella sphenisci*, has been cultured. It was isolated from the excretions of the Magellan penguin (*Spheniscus magellanicus*^88^) which feed on fish and crustaceans. Uncultured members of the genus *Proteocatella* have been described from wastewater, rivers and drinking water and gut microbiota^89–91^. However, the genus also forms a part of the microbiome of carbonized thrombolites found in shallow, subpolar freshwater Laguna Larga in southern Chile^92^. Another interesting fact is that *Proteocatella* spp. is known as a component of feline^93^ and canine^94,95^ dental plaque that may mineralize and become dental calculus^96^. Therefore, species of the genus *Proteocatella* might have supported the calcite precipitation in crayfish carcasses of Exp. 1 and Exp. 3 and might be one of the genera responsible for fossilization. *Oscillibacter* sp. and *Desulfovibrio* sp. were also only present in the experiments with the unsterilized water albeit in low abundances. Both genera have been associated with either mineralization^97–99^ or plaque formation^100^.

It has been widely assumed that anaerobic and especially reducing conditions slow-down soft tissue degradation^29–35^, as they inhibit autolytic enzymes that are responsible for initial decay processes and exclude external scavengers and, thereby, bioturbation^31,38,101^. However, in our experiments, the crayfish tissue was degraded comparably under anaerobic as well as under aerobic starting conditions. This is most probably due to the establishment of anaerobic conditions in all experimental setups within a short time since artificial aeration by incubation on an orbital shaker would have destroyed the carcasses and was therefore not possible^9,14,26,85^. Another explanation for the comparable decay patterns might be insufficient inhibition of autolytic activity in the experiments. In the presence of oxygen, the stomach decomposed after 2 days (Exp. 1, Exp. 2), whereas under anaerobic conditions this organ remained intact until day 3 (Exp. 3, Exp. 4), in contrast to the digestive gland, which decayed later in an oxic surrounding. Before decomposition, the muscles showed a color change from white to pink which was detected earlier under anaerobic than under aerobic conditions, most likely caused by release and diffusion of astaxanthin (AXT), a pigment normally ligated to the α-crustacyanin protein complex in the carapace of living crustaceans^102^. In fact, diffusion of astaxanthin into calcite clusters, which had precipitated at the inner side of the cuticle, has also been observed (Fig. 3b,c).

Other experiments could also detect no^16,38–40^ or rarely a difference in decay rate under various oxygen conditions. Hancy and Antcliffe^41^ even showed that some tissue structures are better preserved in the presence of oxygen. In previous experiments reducing conditions were established by incubation of small organisms with β-mercaptoethanol^34,35,103^. Our results indicate that the concentration of the reducing agent has to be adapted to the body size of the organisms as previous protocols could not guarantee efficient inhibition of autolytic processes.

The microbiome of organisms is individual and thereby, may influence the degradation and preservation differently^35,104^, and, therefore, allows the assumption of an individual conservation potential in nature. However, in these experiments, all animals came from the same tank and the biological replicates exhibited the same tendencies in microbial succession and tissue breakdown. The analysis of the origin of degrading or preserving organisms was complex and most of the dominating bacteria could be found in the tissue as well as in water and sediment. In addition, the analysis of microbial community composition and their influence on decay and preservation of soft tissue is hampered by bacteria that exhibit variable behavior under different environmental compositions, such as the marine decomposer *P. tunicata*^31^. To obtain further information on the behavior of bacteria and their active metabolic pathways, transcriptome analysis should be performed.

In conclusion, a fixed decomposer succession of *Aeromonas*, *Clostridium* and *Acetobacteroides* that closely reproduced the “necrobiome” described for freshwater fish^62^ was observed under different experimental conditions for *Cambarellus diminutus*. The presence of these bacteria was accompanied by the formation of crystalline calcite within the animals and was especially pronounced in the presence of the genus *Proteocatella*.

## Supplementary Information

Supplementary Figures.

Supplementary Tables.

## Data Availability

All raw sequence data related to this study are deposited in the European Nucleotide Archive (ENA) (European Bioinformatics Institute, EMBL-EBI) database a collaboration partner of the International Nucleotide Sequence Database (INSDC), [Study-Accession Number: PRJEB51206]. https://www.ebi.ac.uk/ena/browser/view/PRJEB6609.
