# Supplementary material for: Time-dependent microbial shifts during crayfish decomposition in freshwater and sediment under different environmental conditions
Source: Sci Rep. 2023 Jan 27;13:1539. doi: 10.1038/s41598-023-28713-x (PMC9883499; doi:10.1038/s41598-023-28713-x)
Supplement: Supplementary file 1 — Supplementary Figures. [file 41598_2023_28713_MOESM1_ESM.pdf]

Supplementary Information:

Time-dependent microbial shifts during crayfish decomposition in freshwater and sediment under different environmental conditions

Bastian Mähler[1*], Kathrin Janssen[2*], Mara Iris Lönartz[3,4], Markus Lagos[3], Thorsten Geisler[3], Jes Rust[1] & Gabriele Bierbaum[2]

[1] Section Paleontology, Institute of Geosciences, Rheinische Friedrich-Wilhelms Universität Bonn, 53115 Bonn, Germany

[2]Institute of Medical Microbiology, Immunology and Parasitology, Medical Faculty, Rheinische Friedrich-Wilhelms Universität, 53127 Bonn, Germany

[3]Section Geochemistry, Institute of Geosciences, Rheinische Friedrich-Wilhelms-Universität Bonn, 53115 Bonn, Germany

[4]Institute of Energy and Climate Research (IEK-6): Nuclear Waste Management, Forschungszentrum Jülich GmbH, Jülich, Germany

*Authors contributed equally to this work (correspondence: bastian.maehler@uni-bonn.de; s5kajans@uni-bonn.de)

Systematic zoological position of *Cambarellus diminutus*

**Phylum:**	Arthropoda
**Subphylum:**	Crustacea
**Class:**	Malacostraca
**Order:**	Decapoda
**Family:**	Cambaridae
**Genus:**	*Cambarellus*
**Species:**	*C. diminutus*

Supplementary Figures 1 - 5

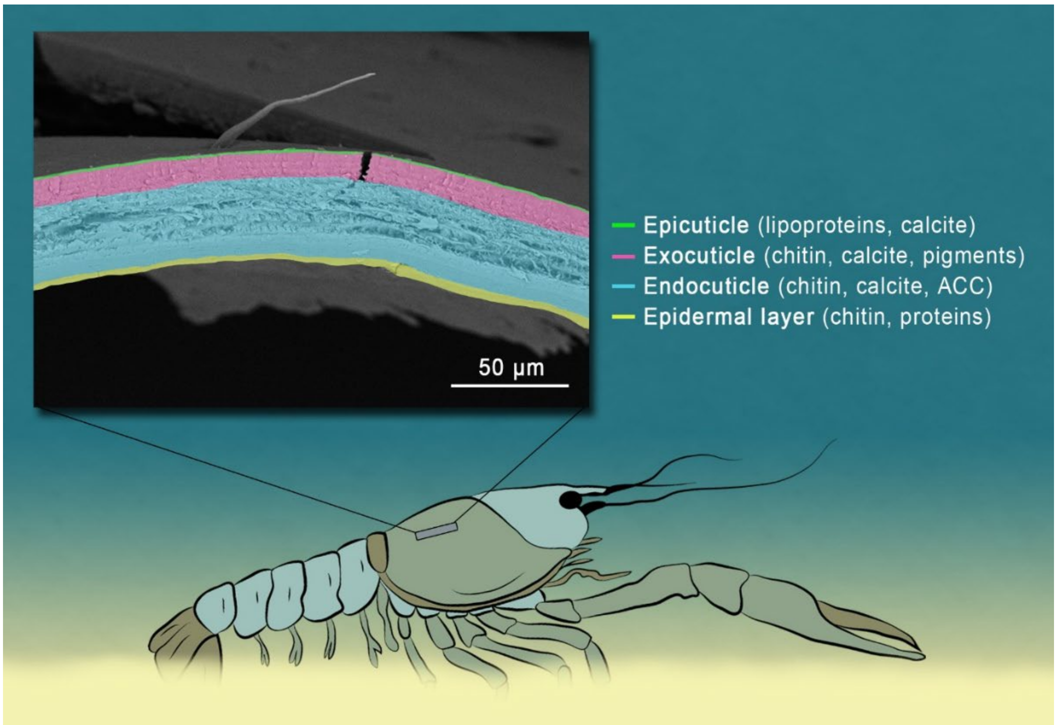

Supplementary Figure 1 General construction of the cuticle of *Cambarellus diminutus*. The box shows a BSE-image of a cross section of a fresh crayfish cuticle with colored layers. **ACC**, amorphous calcium carbonate. (Mähler, 2022).

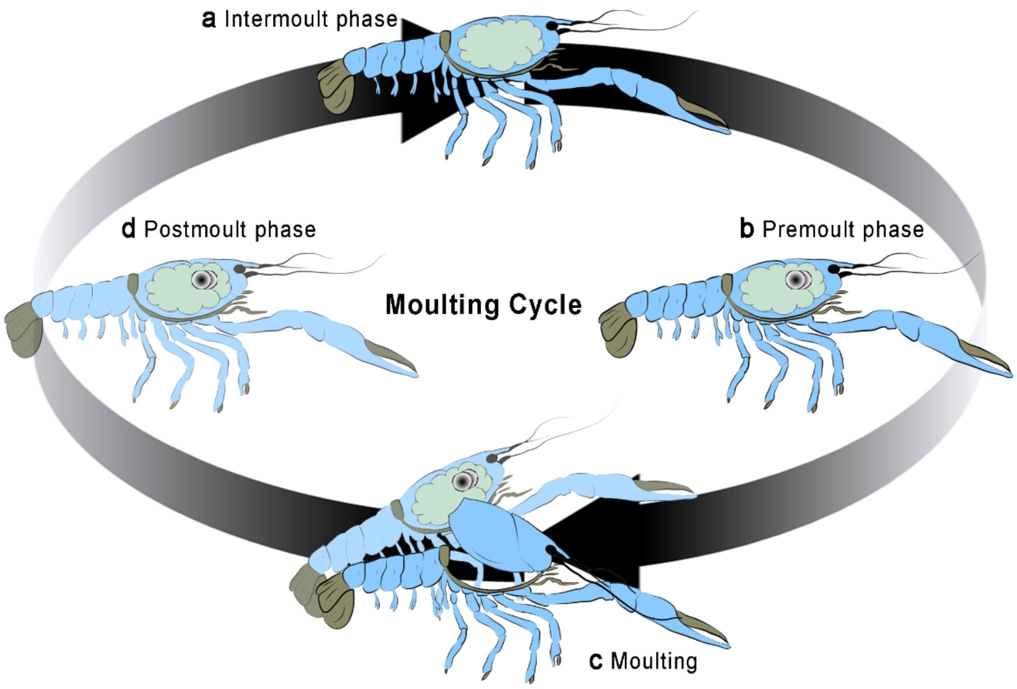

Supplementary Figure 2 Moulting cycle of the freshwater crayfish *Cambarellus diminutus*. **a** Individual with completely hardened cuticle during the intermoult phase. **b** Individual with one pair of gastroliths inside the cardiac stomach wall during the premoult phase. **c** Crayfish individual is slipping out of the old cuticle during the moulting process. **d** Individual with a not fully hardened cuticle right after the moulting. (Mähler, 2022).

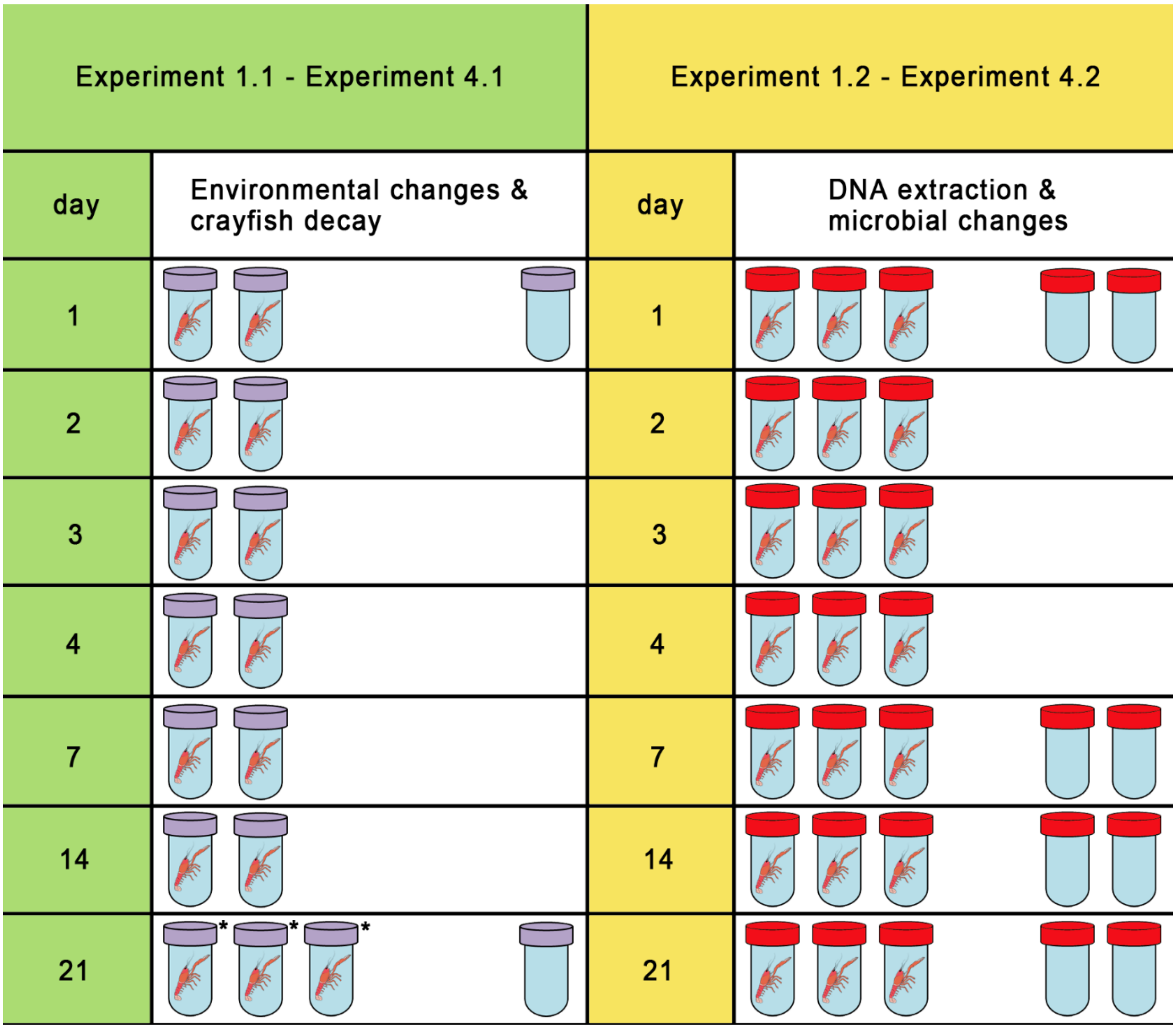

Supplementary Figure 3 Additional information to the experimental setup. Exp. 1.1 – Exp. 4.1 were started simultaneously to Exp. 1.2 – Exp. 4.2. **Exp. 1.1 – Exp. 4.1** Each experiment was conducted with 15 crayfish individuals and 2 blank samples (60 individuals and 8 blanks in complete). **Exp. 1.2 – Exp. 4.2** Each experiment was conducted with 21 crayfish individuals and 8 blank samples (84 crayfish individuals and 32 blanks in complete). ***Individuals were scanned via μ-CT on day 1 – 4 and on day 7, 14 and 21.**

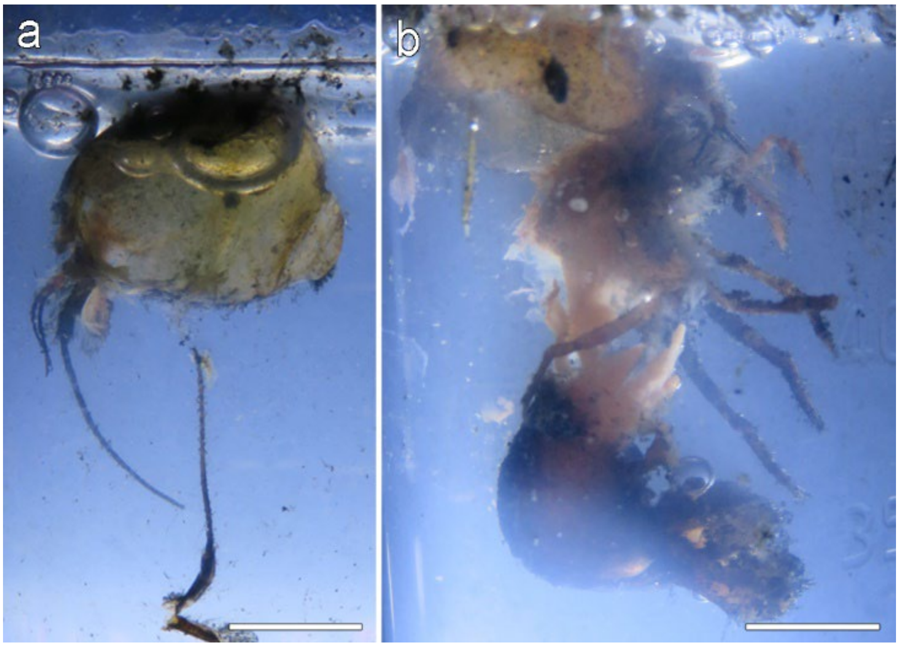

Supplementary Figure 4 Remains of decomposing crayfish on day 24. **a** Floating cephalothorax of crayfish specimen E1-21.1 with gas bubbles inside. **b** Partly detached cephalothorax floating and holding the rest of the dangling carcass of crayfish specimen E1-21.3. *Scale bar* 1 cm.

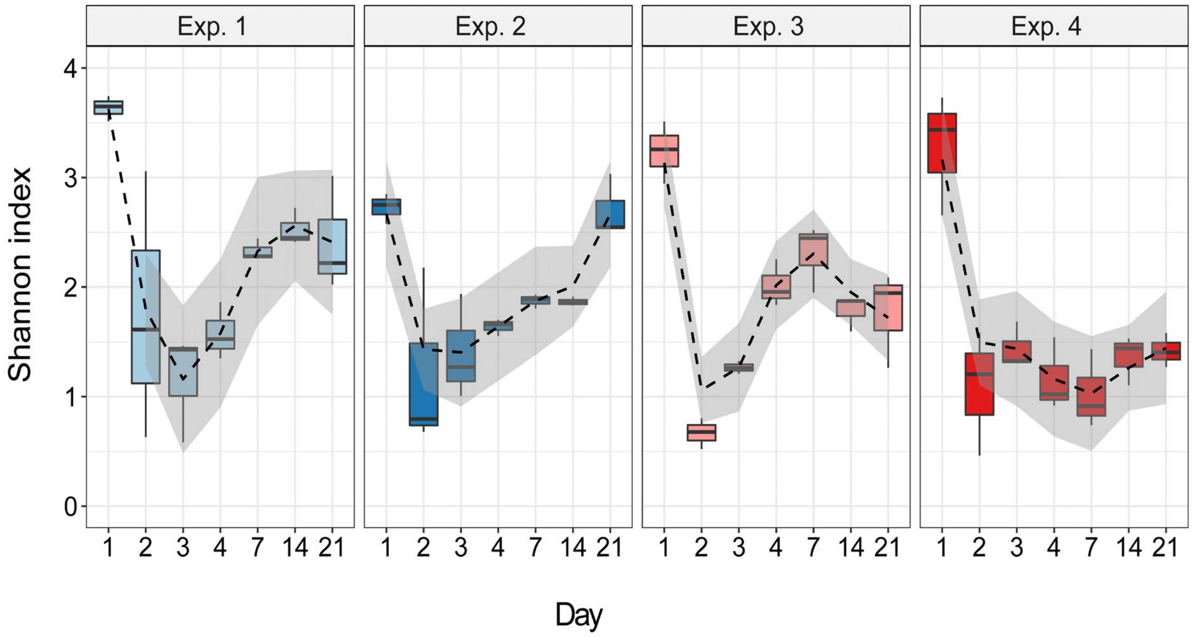

Supplementary Figure 5 Comparison of the time-dependent shift in alpha diversity measured via shannon index during the four experiments.

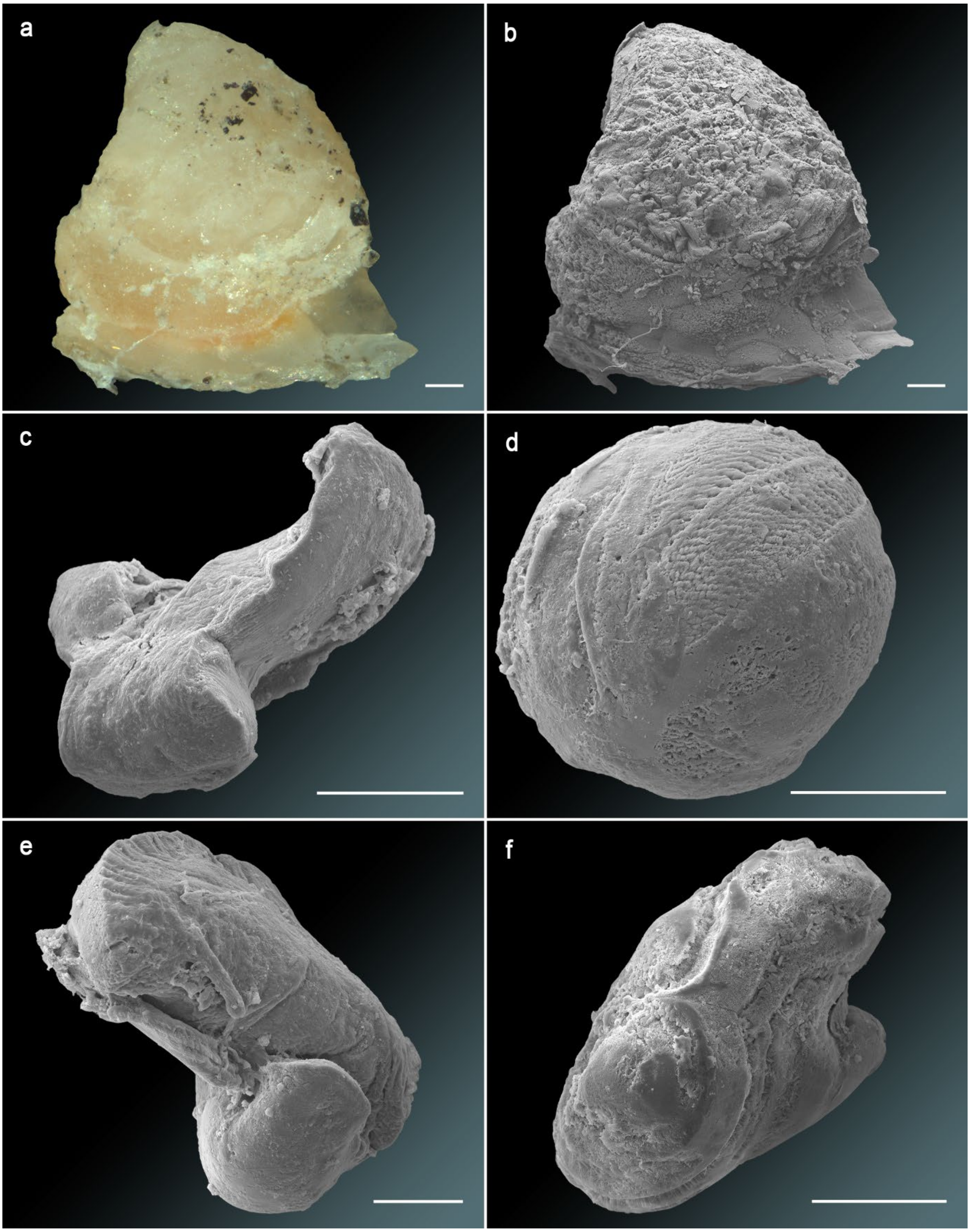

Supplementary Figure 6 Stereoscopic image and SEM images of calcite clusters found in specimen E1-21.2 at the end of Exp. 1. **a** Stereoscopic image of a conical calcite cluster. **b** SEM image of the cluster shown in 3a. **c-f** SEM images of diverse calcite clusters. *Scale bar* 100 µm.
